# Skin-specific mechanisms of body fluid regulation in hypertension

**DOI:** 10.1042/CS20220609

**Published:** 2023-02-07

**Authors:** Jun Yu Chen, Khai Syuen Chew, Sheon Mary, Philipp Boder, Domenico Bagordo, Gian Paolo Rossi, Rhian M. Touyz, Christian Delles, Giacomo Rossitto

**Affiliations:** 1 School of Cardiovascular & Metabolic Health, University of Glasgow, U.K.; 2 Emergency Medicine and Hypertension, DIMED, Università degli Studi di Padova, Italy; 3 Research Institute of McGill University Health Centre, McGill University, Montreal, Canada

**Keywords:** homeostasis, hypertension, skin, sodium, sweat, water

## Abstract

Increasing evidence suggests excess skin Na^+^ accumulation in hypertension; however, the role of skin-specific mechanisms of local Na^+^/water regulation remains unclear. We investigated the association between measures of sweat and trans-epidermal water loss (TEWL) with Na^+^ content in the skin ([Na^+^]_skin_) and clinical characteristics in consecutive hypertensive patients. We obtained an iontophoretic pilocarpine-induced sweat sample, a skin punch biopsy for chemical analysis, and measures of TEWL from the upper limbs. Serum vascular endothelial growth factor-c (VEGF-c) and a reflectance measure of haemoglobin skin content served as surrogates of skin microvasculature. In our cohort (*n* = 90; age 21–86 years; females = 49%), sweat composition was independent of sex and BMI. Sweat Na^+^ concentration ([Na^+^]_sweat_) inversely correlated with [K^+^]_sweat_ and was higher in patients on ACEIs/ARBs (*P* < 0.05). A positive association was found between [Na^+^]_sweat_ and [Na^+^]_skin_, independent of sex, BMI, estimated Na^+^ intake and use of ACEi/ARBs (*P*_adjusted_ = 0.025); both closely correlated with age (*P* < 0.01). Office DBP, but not SBP, inversely correlated with [Na^+^]_sweat_ independent of other confounders (*P*_adjusted_ = 0.03). Total sweat volume and Na^+^ loss were lower in patients with uncontrolled office BP (*P*_adjusted_ < 0.005 for both); sweat volume also positively correlated with serum VEGF-c and TEWL. Lower TEWL was paralleled by lower skin haemoglobin content, which increased less after vasodilatory pilocarpine stimulation when BMI was higher (*P* = 0.010). In conclusion, measures of Na^+^ and water handling/regulation in the skin were associated with relevant clinical characteristics, systemic Na^+^ status and blood pressure values, suggesting a potential role of the skin in body-fluid homeostasis and therapeutic targeting of hypertension.

## Introduction

The kidney has traditionally been regarded as the main regulator of sodium (Na^+^) and blood pressure (BP) homeostasis. In recent years, our understanding of these aspects expanded to include extrarenal sites of Na^+^ and water handling, with evidence of tissue Na^+^ accumulation and extracellular volume plasticity [1–3]. Peripheral tissues and in particular skin were found to act as a depot for excess Na^+^, with feedback mechanisms in place to ensure its drainage and ultimately whole-body Na^+^ balance [4,5]. Additionally, multiple systemically-acting neurohormonal axes and adaptations of the vascular system contribute to the maintenance of a steady state [6]. At odds with the abundant evidence for a systemic storage and regulation, the sole urinary route of Na^+^ excretion has dominated the focus of researchers for over a century. The relevance of other routes in relation to the pathophysiology and opportunity for treatment of hypertension have largely been neglected likely because of complexities regarding their investigation and the general perception of a quantitatively trivial role compared to the urinary system.

In particular, the skin has already been shown to be a site of lymphatic-mediated peripheral control of Na^+^ status, and of BP, accordingly [4,5]. Additional evidence from psoriatic mice recently suggested that the skin microvascular tone could modulate arterial BP per se [7]. Most relevant to our hypotheses, with its large surface area skin serves as a barrier that preserves the *milieu interieur* [8,9]: modulation of the exchange of Na^+^ and water with the external environment, mainly via sweat and trans-epidermal evaporation of water, could be important in their net loss and, ultimately, in blood pressure control. Surprisingly, there is a paucity of information on reduced sweat sodium loss in hypertensive subjects compared with controls, suggesting an inverse relationship between blood pressure and sweat Na^+^ concentration ([Na^+^] _sweat_) [10]. Additionally, generalizability of these data were questioned in relation to the unclear salt-status of the hypertensive population, treated in almost all cases with diuretics and recruited in a site near the equator (Maracaibo, Venezuela) [11]. More recently, in young healthy individuals, [Na^+^]_sweat_ was associated with salt intake and Na^+^ accumulation in tissues measured by ^23^Na-MRI [12], thus supporting the hypothesis that local skin mechanisms in humans may contribute to the regulation of sodium/fluid balance, and blood pressure accordingly. Direct evidence in this direction is lacking in hypertensive patients, but recent evidence from rodent models of skin disease [13], which appeared to directly elevate BP via water-preserving mechanisms, provides initial ground to this contention.

Our study aimed to investigate the association between sweat and trans-epidermal water loss (TEWL) measures, reflective of local skin Na^+^ and water exchange, respectively, with (1) tissue Na^+^ concentration in the skin ([Na^+^]_skin_), and (2) clinical and biochemical characteristics, in a real-life hypertensive cohort.

## Methods

The protocol for the cross-sectional S_2_ALT (Skin Sodium Accumulation and water baLance in hyperTension) study was approved by the West of Scotland Research Ethics Committee 3 (ref. 18/WS/0238) and Greater Glasgow and Clyde NHS Research and Development (ref. GN18CA634). The study was conducted in compliance with the Declaration of Helsinki. All patients provided written informed consent.

### Study design and protocol

Adult hypertensive patients were recruited from the Blood Pressure (BP) Clinic, Queen Elizabeth University Hospital, Glasgow, between March and July 2019. Patients were invited to participate through information leaflets and invitation letters sent 10 days before their routine clinic appointments. Exclusion criteria included pregnancy, skin conditions such as eczema or psoriasis and, for sweat collection only, implanted pacemakers or implantable cardiac defibrillators. On the day of their scheduled appointment (between 9.00 am and 4.30 pm), those consenting to take part had anthropometric (body height and weight) and routine office BP measures taken as per current guidelines [14,15] with an automated oscillometric device; pulse pressure was calculated as systolic BP−diastolic BP. For this study, uncontrolled BP was defined as office systolic BP ≥ 140 mmHg or diastolic BP ≥ 90 mmHg.

Relevant comorbidities and ongoing medications were recorded. On the same occasion we collected a pilocarpine-induced sweat sample, EDTA-plasma and serum samples (for NT-proBNP and for Na^+^, urea, creatinine and serum VEGF-c, an angiogenic factor implicated in BP control by inducing lymphatic vessel growth via VEGFR3 and increased eNOS expression in blood vessels via VEGFR2 [4], which is reduced in ageing, hypertension and CKD [16], and increased by salt intake [17]). We also measured TEWL and a reflectance measure of haemoglobin (Hb) skin content, at an unstimulated and a pilocarpine-stimulated forearm site, and lastly, we administered a short questionnaire to estimate sodium intake.

Study participants were also offered an optional skin punch biopsy. Part of the data from the subgroup of patients who consented to undergo the biopsy (*n* = 76), including skin biochemical analysis and relevant blood pressure, estimated Na^+^ intake and NT-proBNP measures, have already been reported when we addressed the nature of tissue Na^+^ accumulation [3].

### Pilocarpine-induced sweat sampling

Immediately before the clinic visit, upon consent and exclusion of an implanted pacemaker/cardioverter-defibrillator, a small area of the skin on the flexor part of the forearm of the study participants was cleaned with distilled Milli-Q water. The validated Macroduct Sweat Collection System (Webster Sweat Inducer Model 3700; ELITechGroup, Puteaux, France) was used to induce iontophoresis using pilocarpine gel pads (Pilogel® Iontophoretic Discs, ELITech Group) for 5 min (1.5 mA) [18]. After removal of the pilocarpine pads and gentle cleaning with sterile swabs, a dedicated plastic coil (Macroduct® no-dye collectors, ELITech Group) was attached on the stimulated arm with straps and secured with Parafilm® wrapping. After 30 – 40 min, the sweat collected in the coil was transferred to a 0.2 ml microtube, immediately frozen in dry ice and stored at −80 °C in airtight tubes until biochemical analysis. At the time of the analysis, samples were thawed to measure the sweat volume with a calibrated micropipette and appropriately diluted to determine Na^+^ and K^+^ concentrations (mmol/l) by flame photometry. Total Na^+^ or K^+^ sweat content was calculated as concentration × volume. As per manufacturers and guidelines recommendations, samples < 15 μl were considered unreliable and excluded from the analysis.

### Trans-epidermal water loss

Before blood collection and skin biopsy and following a ≥ 20-min period of acclimatization in a temperature-controlled environment (20 – 21 °C) and ≥ 5 min of quiet sitting, trans-epidermal water loss (TEWL, [19]) were assessed from the flexor portion of the forearm contralateral to the site of sweat collection using a Tewameter® TM300 probe (Courage & Khazaka GmbH, Cologne, Germany). The probe estimates TEWL through two pairs of temperature and humidity sensors using Fick’s diffusion law. Readings were automatically stopped and recorded by an MPA 580 system and dedicated software when a ≤ 0.1 standard deviation was reached.

### Mexameter

A Mexameter® MX 18 (Courage & Khazaka GmbH, Cologne, Germany) was connected to the same MPA system for additional skin physiology-related assessment. The calibrated probe is pressed on the measurement site for ∼1 s and quantifies haemoglobin (Hb) content in the skin by reflectance, expressed as Erythema Index (arbitrary units, AU; [20,21]). The difference between the Hb content in the skin area that underwent pilocarpine stimulation with that of the contralateral limb in the same flexor portion of the forearm was obtained. Each value was measured at least in duplicate and averaged. Δ skin Hb content was used as a surrogate of acetylcholine-mediated vasodilatation [22], based on previously demonstrated excellent correlation with changes in blood flow measured with a laser doppler flowmeter [23].

### Salt intake questionnaire

A short, validated questionnaire [24] was administered to patients while waiting for their scheduled visit. Calculation was made as reported [24]. Briefly, it included 42 food items with six possible consumption frequency responses: never; one to three times per week; four to six times per week; once a day; twice a day; and three plus times a day. Predefined absolute amounts of sodium per serving size per specific item, according to MRC Food Composition Tables were multiplied by the consumption frequency factor each individual reported and added up to a total weekly Na^+^ intake for each subject, later divided by seven to estimate daily Na^+^ intake. For simplicity, the absolute amounts of Na per serving for each food category was divided by 50 mg Na^+^ units and rounded to the nearest integer. The frequency factor in the weekly calculation was taken as the value midway between the upper frequency value of one category and the lower of the next (i.e., 0, 2, 5, 7, 14, and 21).

Missing questionnaire data was missing at random (Little’s MCAR test, *P*=0.401). For five subjects, with over half of the questionnaire responses missing, calculations of weekly scores were considered to be unreliable and excluded. Twenty-eight additional subjects had ≤ 10% missing responses (13/28 had only 1 missing item) and median substitution was used for imputation and calculation of weekly scores. Overall, results without imputation were identical.

### Skin punch biopsy and biochemical analysis

At the time of pilocarpine stimulation, a Na^+^/K^+^-free lidocaine-based topical anaesthetic cream (LMX4, Ferndale Pharmaceuticals Ltd) was applied on the outer upper contralateral arm, approximately halfway between the elbow and shoulder [3]. After collection of sweat samples and TEWL measurements and after cleaning the skin with cotton gauze pads and Na^+^/K^+^-free 70% alcohol wipes, skin punch biopsies were performed on the anaesthetized site with a disposable instrument (3 – 4mm blade diameter; Kai Medical). The sample was immediately placed into a pre-cooled Eppendorf tube, frozen in dry ice and subsequently stored at −80 °C until tissue analysis.

The frozen skin samples were transversally cut into a superficial layer, including the epidermis and the immediately adjacent superficial dermis (ESD), and a deeper dermal layer in a cold room to prevent water evaporation. Tissue water content was gravimetrically assessed on a five decimal (0.00001 g) scale (Ohaus, DV214CD), and calculated as the difference between wet weight and dry weight after complete sample desiccation at 65 °C for > 40 h. Dried samples were digested in HNO_3_, and Na^+^ and K^+^ content was quantified by flame photometry, as reported [3]. For this study, skin Na^+^ concentration ([Na^+^]_skin_; mmol/l) refers to the ESD Na^+^ concentration, which is unaffected by the confounding impact of deeper dermal fat, is highly correlated with ESD water and circulating NT-proBNP and serves as a surrogate of the extracellular volume and total body Na^+^ [3].

### Biochemistry

Blood samples were immediately processed and stored at −80 °C until biochemical analysis. Sodium, urea, creatinine, NT-proBNP and spot urine albuminuria were measured on a Cobas analyser (Roche Diagnostics GmbH, Mannheim) by ion-selective electrode, Urease/GLDH, Jaffè and electrochemiluminescence immunoassay (Elecsys® proBNP), respectively. The CKD-EPI formula was used to calculate the eGFR. VEGF-c was measured in serum (Quantikine ELISA, R&D).

### Statistical analysis

Statistical analysis was performed using Prism (GraphPad Software) and SPSS (IBM).

Categorical variables are presented as absolute numbers and percentages and compared by χ² test. Student’s *t*-test for normally distributed variables (presented as mean ± SD or, graphically, as mean [95% CI]) or Mann–Whitney test for non-normally distributed variables (presented as median [interquartile range] or, graphically, as median [95% CI]) were used for comparisons. Outliers were automatically identified by ROUT method (*Q* = 1%) and excluded from analysis but reported on the respective figures. Correlations were ascertained by Pearson’s test, upon appropriate transformation of skewed variables to attain normal distribution, or Spearman if normality was not attained. Univariable and multivariable linear regression models were estimated (including ANCOVA) and results were presented as B coefficients (95% CI). Analysis of variance for one dependent variable by one or more factors and/or variables was performed by UNIANOVA in SPSS. The α level was set at 0.05, and all statistical tests were two-tailed (*P<0.05, **P<0.01, ***P<0.001, ****P<0.0001).

## Results

A total of 90 patients, almost exclusively Caucasian (98%), were included. Males (*n* = 46) and females (*n* = 44) were equally represented. Their characteristics, notable for a high prevalence of obesity and a broad age range (21 – 86 years), are presented in Table 1. Of all participants, 74% had office BP values above 140/90 mmHg (*uncontrolled hypertension*), with a significantly higher prevalence in males compared with females (85% vs 62%, *P* = 0.015) despite a higher use of first-line antihypertensive medications. Otherwise, males and females were reasonably balanced, with the exception of the expected biochemical differences in plasma urea and creatinine [25].

**Table 1:** Characteristics of patients, *n* = 90

Variables	All (*n*=90)	Females	*P*	Males
**Females**	**44 (48.9%)**	–		–
**Age** (years)	**56 ± 16**	55 ± 17	0.552	57 ± 14
**BMI** (kg/m^2^)	**29.8 (26.9–35.2)**	30.3 (26.3–36.7)	0.370	29.7 (27.7–33.0)
**BMI class**				
**Normal weight**	**10 (11.2%)**	6 (14%)	0.516	4 (8.7%)
**Overweight**	**36 (40.4%)**	15 (34.9%)		21 (45.7%)
**Obese**	**43 (48.3%)**	22 (51.2%)		21 (45.7%)
**Office SBP** (mmHg)	**148 ± 20**	148 ± 24	0.493	148 ± 15
**Office DBP** (mmHg)	**89 ± 11**	87 ± 13	0.239	90 ± 10
**Office HR** (beats per minute)	**74 (65–87)**	82 (68–89)	0.503	73 (65–83)
**Uncontrolled HTN**	**65 (73.9%)**	26 (61.9%)	**0.015**	39 (84.8%)
**Number of anti-HTN medications**	**1 (0–2)**	1 (0–2)	**0.008**	2 (1–2)
**0**	**23 (25.6%)**	14 (31.8%)	0.081	9 (19.6%)
**1**	**23 (25.6%)**	15 (34.1%)		8 (17.4%)
**2**	**27 (30%)**	9 (20.5%)		18 (39.1%)
**3**	**12 (13.3%)**	5 (11.4%)		7 (15.2%)
**≥4**	**5 (5.6%)**	1 (2.3%)		4 (8.7%)
**ACEi/ARB**	**68 (75.6%)**	28 (63.6%)	**0.010**	40 (87%)
**CCB**	**49 (54.4%)**	19 (43.2%)	**0.036**	30 (65.2%)
**Diuretic**	**38 (42.2%)**	13 (29.5%)	**0.017**	25 (54.3%)
**BB**	**21 (23.3%)**	9 (20.5%)	0.528	12 (26.1%)
**MRA**	**11 (12.2%)**	4 (9.1%)	0.375	7 (15.2%)
**AB**	**14 (15.6%)**	7 (15.9%)	0.928	7 (15.2%)
**Obesity**	**44 (49.4%)**	23 (53.5%)	0.460	21 (45.7%)
**Diabetes mellitus**	**12 (13.5%)**	4 (9.3%)	0.264	8 (17.4%)
**Dyslipidaemia**	**46 (51.1%)**	17 (41.5%)	**0.044**	29 (63%)
**eGFR < 60 (ml/min/1.73 m^2^)**	**8 (10.5%)**	4 (9.1%)	0.551	6 (13%)
**Na^+^ intake** (g/d; questionnaire)	**2.79 (2.04-3.66)**	2.63 (1.89–3.30)	0.186	2.96 (2.13–4.10)
**s-Na^+^**	**140 (139-142)**	140 (139–142)	0.225	140 (139–141)
**s-Urea** (mmol/l)	**5.2 (4.6–6.5)**	4.9 (4.0–6.1)	**0.010**	5.5 (4.9–7.2)
**s-Creatinine** (umol/l)	**73 (63–86)**	65 (55–74)	**<0.001**	83 (71–95)
**NT-pro-BNP** (pg/ml)	**71.8 (41.4-167.6)**	87 (46–152)	0.227	59 (30–216)
**s-VEGFc** (ng/ml)	**13.2 ± 3.3**	12.9 ± 3.4	0.230	13.5 ± 3.1

Qualitative data presented as *n* (%). Quantitative data presented as mean ± SD or median (interquartile range), as appropriate. Abbreviations: AB, α blockers; ACEi/ARB, ACE inhibitors or angiotensin receptor blockers; BB, β-blockers; BMI, body mass index; CCB, calcium channel blockers; DBP, diastolic blood pressure; HR, heart rate; HTN, hypertension; MRA, mineralocorticoid antagonists; s, serum; SBP, systolic blood pressure. Uncontrolled HTN = SBP ≥ 140 and/or DBP ≥ 90 mmHg.

### Sweat analysis

Sweat collection was contraindicated or declined in four cases; in six more cases, the yield was ≤7 μl, which was remarkably lower than all other samples and lower than the minimum reliable volume recommended by guidelines (15 μl), likely suggestive of inadequate collection [18,26]. These cases were excluded from the analysis.

In the 80 valid sweat samples, [Na^+^]_sweat_ (30.5 [24.4 – 42.3] mmol/l) inversely correlated with [K^+^]_sweat_ (ρ: = −0.230, *P* = 0.041). No simple linear association was found between [Na^+^]_sweat_ and sweat volume (*V*_sweat_), as a surrogate for rate. However, after exclusion of a group of patients clustering at one corner of the [Na^+^]_sweat_ × *V*_sweat_ plot, with high sweat rates but low tonicity, a third order polynomial consistent with expected sweat physiology [27] could be fitted (*P* < 0.0005, Supplementary Figure S1). These ‘high hypotonic sweaters’ (HHS, *n* = 23) featured a younger age (51 ± 14 vs 58 ± 16 years, *P* = 0.039), higher BMI (32.8 [28.5 – 36.8] vs 29.3 [26.4 – 34.5] kg/m^2^, *P* = 0.045), higher serum VEGF-c (14.4 ± 2.6 vs 12.8 ± 3.4 ng/ml, *P* = 0.039) and a trend for higher DBP (93 ± 11 vs 87 ± 11 mmHg, *P* = 0.055) compared with the other participants.

No difference in sweat volume or composition was found between sexes. Patients treated with angiotensin system blockers, i.e. angiotensin-converting-enzyme inhibitors (ACEi) or angiotensin receptor blockers (ARB), had higher [Na^+^]_sweat_ compared with those who were not (32.8 [26.0 – 45.8] vs 25.7 [17.7 – 36.7] mmol/l, *P* = 0.007), even when the analysis was limited to subjects taking no other antihypertensive medications (*n* = 12, 40.4 [30.9 – 52.8] vs *n* = 8, 27.2 [20.5 – 39.6] mmol/l, *P* = 0.05). No similar interaction with either sweat composition or volume was found for diuretics or calcium channel blockers; for other classes of antihypertensive medications, numbers were too limited for robust conclusions.

**Figure 1:**
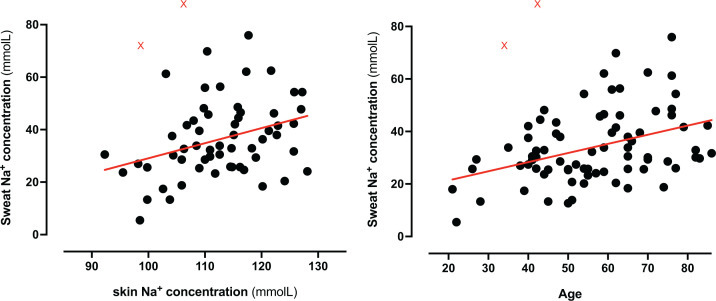
Association of sweat Na ^+^ concentration with skin Na ^+^ and age Association of sweat Na ^+^ concentration with skin Na ^+^ and age Tissue Na^+^ concentration in the skin and age are positively correlated with sweat Na ^+^ (ρ = 0.302, *P* = 0.019 and ρ = 0.341, *P* = 0.002, respectively; linear regression lines are depicted in red). Red X = automatically identified outliers (ROUT = 1%); after their removal, statistics revealed ρ = 0.367, *P* = 0.001 and ρ = 0.345, *P* < 0.01, respectively.

Correlations between sweat volume and composition with relevant clinical covariates, including Na^+^ concentration in the epidermis/superficial dermis as a surrogate for skin extracellular volume and, indirectly, of body Na^+^ [3,28], are reported in Supplementary Table S1. Of note, both age and [Na^+^]_skin_ positively correlated with [Na^+^]_sweat_ (Figure 1). The association between [Na^+^]_skin_ and [Na^+^]_sweat_ was independent of sex, BMI, estimated Na^+^ intake and use of ACEi/ARB (*P*_adjusted_ = 0.015; after removal of statistical outliers as per Figure 1, *P*_adjusted_ = 0.003), but not age. No remarkable differences were observed between sexes, except a mild but significant correlation between NT-proBNP and [Na^+^]_sweat_ in males only (Spearman, ρ = 0.329, *P* = 0.043). In the aforementioned HHS subgroup, the association between age and [Na^+^]_sweat_ was lost; however, [Na^+^]_sweat_ inversely correlated with skin K^+^ content (ρ = 0.499, *P* = 0.049) and concentration (ρ = 0.534, *P* = 0.040), in contrary to the remaining cohort (*P* = 0.489).

**Figure 2:**
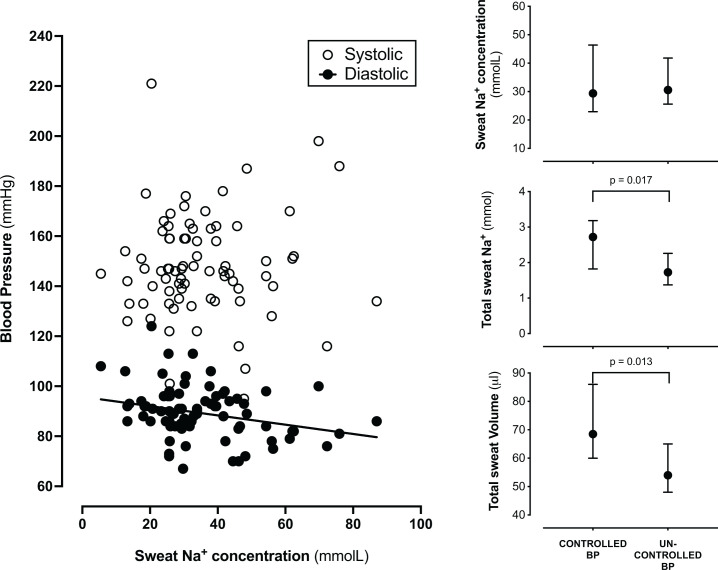
Sweat measures in relation to blood pressure values and control. Sweat Na^+^ concentration is inversely and independently associated with diastolic BP. Office BP control is associated with higher total sweat Na^+^ and total sweat volume (data presented as median [95% CI]).

Office diastolic, but not systolic, BP inversely correlated with [Na^+^]_sweat_ (ρ = −0.286, *P* = 0.012; Figure 2). The association was independent of sex, BMI, estimated Na^+^ intake and use of ACEi/ARB (beta for log10-transformed [Na^+^]_sweat_ (mmol/l) = −14.1 (95% CI: −25.6, −2.6); *P*_adjusted_ = 0.030). Patients with uncontrolled office BP had [Na^+^]_sweat_ values similar to those with BP below 140/90 mmHg, despite their older age (59 ± 15 vs 49 ± 16 years, *P* = 0.392), the other main characteristics being similar between groups except for prevalence of dyslipidaemia, as reported in Supplementary Table S2. However, along with lower circulating VEGFc and borderline higher NT-proBNP concentrations, their total sweat volume and total Na^+^ loss were lower regardless of age, sex, BMI, estimated Na^+^ intake, and use of ACEi/ARB (*P* < 0.01 and *P*_adjusted_ < 0.005 for both). At variance with these patients, in the group with controlled office BP values, [Na^+^]_sweat_ was remarkably associated with BMI (ρ = −0.738, *P* < 0.001), and sweat volume with NT-proBNP (ρ = −0.545, *P* < 0.019).

### Trans-epidermal water loss and surrogates of skin microvasculature

TEWL measures at rest were not associated with any relevant clinical feature, including age, sex, BMI, diabetes or CKD status, estimated Na^+^ intake, biochemical signatures or the use of different medications. Similarly, no correlation with office SBP or DBP was identified (Supplementary Table S1). However, TEWL positively correlated with sweat volume (ρ = 0.292, *P* = 0.020) and surrogate measures of the skin microvasculature, serum VEGF-c (ρ = 0.261, *P* = 0.029) and, with borderline significance, a reflectance measure of Hb skin content (ρ = −0.191, *P*=0.077). Of note, the absolute value of this parameter assessed after local cholinergic stimulus, and its change vs baseline (Δ skin Hb content) showed a robust negative correlation with BMI (ρ = −0.352, *P* = 0.002 and ρ = −0.283, *P* = 0.013, respectively); the association was independent of age, sex, estimated Na^+^ intake, office BP control, [Na^+^]_skin_ and plasma Na^+^ (β = −4.35 [−7.60 to -1.09], *P* = 0.010). Plasma Na^+^ positively correlated with this acetylcholine-induced hyperaemic response (ρ = −0.284, *P* = 0.009), but the association disappeared after correction for BMI.

## Discussion

The results of our study overall suggest that modulation of sweat and TEWL, via neurohormonal and microvascular control, could play an active role in the systemic regulation of body Na^+^ and water in human hypertension.

Despite the role of whole-body fluids and electrolytes in determining blood pressure [29], kidneys have traditionally been considered the sole relevant site of regulation. Previously, only one study reported a reduced sweat Na^+^ excretion in hypertensive subjects compared to controls and an inverse correlation between BP and [Na^+^]_sweat_ in both groups, with a steeper association in the former [10]. However, the severity of hypertension, the generous use of diuretics for its treatment and concerns regarding sweat rate in the equatorial study setting casted doubts on those results [11]. Our study, conducted in a real-life variegated population of hypertensive patients and unaffected by the above limitations, provides support to those conclusions.

In our patients we observed results consistent with sweat gland physiology, whereby sweat rate is a fundamental determinant of its tonicity: at low rates of production, the nearly isotonic primary fluid is made progressively hypotonic by ductal Na^+^ reabsorption toward the skin surface, while at very high rates this time-dependent process is impeded and the resulting [Na^+^]_sweat_ is higher. However, we also identified a subgroup, labelled as HHS and characterized by younger age and higher BMI, which showed high reabsorption of Na^+^ despite high sweating rates. This observation could be consistent with the Na^+^-retentive state typical of obesity [30–32] but could also reflect a compensatory excretion of excess free water generated by higher metabolic rates. Of note, previous studies conducted on obese subjects during exercise, showed no significant differences in sweat Na^+^ concentration [33], despite markedly higher sweat rates, compared with controls [33,34].

Our results also suggest that ductal Na^+^ reabsorption retains sensitivity to the renin–angiotensin–aldosterone system (RAAS) in hypertensive patients. Previous research in healthy subjects showed that [Na^+^]_sweat_ is reduced by aldosterone [35], which promotes ductal Na^+^ reabsorption, and is negatively associated with plasma aldosterone concentration and renin activity in relation to Na^+^ intake [12]. On the two extremes of a spectrum, Jerome W. Conn reported a low [Na^+^]_sweat_ in the early description of the syndrome named after him [36], while patients with pseudo-hypoaldosteronism type I exhibit salt loss from all aldosterone target organs including sweat glands in the skin [37]. In our patients, use of ACEi or ARB – but not calcium channel blockers or diuretics – was associated with higher [Na^+^]_sweat_. This suggests a reduced local impact of the RAAS and the local effectors likely include the mineralocorticoid receptor [38,39] and the epithelial Na channel (ENaC [40], Supplementary Figure S2). Additional skin determinants of the individual heterogeneity on sweat rates, concentrations and, ultimately, blood pressure control, including the role of local RAAS [41,42], remain largely unknown; of note, black subjects, whose blood pressure is known to be particularly salt-sensitive, have thrifty sweating patterns characterized by low sweat rates [43].

One important finding of our study is the robust association between [Na^+^]_sweat_ and a direct measure of Na^+^ in the tissues, i.e. [Na^+^]_skin_ as previously described [3]. In the context of the highly variable degree of salt retention that different hypertensive patients exhibit upon salt loading [44], it is tempting to speculate that counterregulatory mechanisms favouring excess Na^+^ loss in sweat would kick in when excess body Na^+^ is sensed and that sweat glands, in parallel to kidneys, are part of this response. This is in keeping with the sweat changes induced by different Na^+^ intakes in a study on healthy subjects [12]: our estimation of Na^+^ intake by means of questionnaires likely lacked the necessary precision and statistical power to identify similar correlations; however, as for our skin biochemical data, ^23^Na-MRI-assessed muscle Na^+^ content correlated with [Na^+^]_sweat_ in that cohort. No major differences between sexes emerged from either study. On the contrary, we identified an independent and physiologically relevant association between [Na^+^]_sweat_ and ageing, a condition known to feature total body Na^+^ excess[45]: of note, the RAAS adaptations to changes in Na^+^ homeostasis, possibly including the stimulus for sweat ductal Na^+^ reabsorption, are blunted with older age [46].

Regardless of RAAS blockade, sex, BMI or estimated Na^+^ intake, the patients that presented with uncontrolled office BP showed lower sweat Na^+^ loss upon pilocarpine stimulation. There are possible confounders to this finding, including the nature of office BP values [47] or the different lipid profiles between controlled and uncontrolled hypertensives, which could modulate Na^+^ avidity via excess aldosterone biosynthesis [48,49]. However, consistency with previous reports [10] supports the contention that an impairment in sweat Na^+^ excretion, as extensively demonstrated for renal routes [50], could contribute to hypertension if also this route were quantitively relevant to whole-body total Na^+^ balance. Only whole-body sweat collections can reliably inform on this matter, but methods are cumbersome and not devoid of biases or difficulties in ensuring complete recovery [51]. Heer et al suggested that skin sodium losses could be neglected during long-term experiments, but the conclusions were based on three subjects constrained in a metabolic ward for 48 h [52]. Their sweat Na^+^ loss rates were markedly lower than those reported in other studies including different levels of physical activity [53,54], which resulted in in a loss of 29 mmol Na^+^ after 90 min at low intensity, increasing to 68 mmol with moderate intensity, neither of which is negligible in everyday life [54]. Moreover, the size of the sweat glands varies as much as fivefold among different persons, which largely correlates with the individual difference in the rate of sweating [55]. All in all, we suggest that the combination of different sweat rates, based on individual characteristics or the degree of physical activity, and different sodium ductal handling [56] as observed in our patients with uncontrolled BP or the HHS subgroup but also across healthy individuals [12], could contribute to sodium homeostasis and blood pressure control, accordingly. This suggestion seems at odds with the orthostatic hypotension observed in patients with anhidrosis [57]; however, this condition is secondary to autonomic dysfunction, in which orthostatic hypotension does not exclude hypertension [58], and in the vast majority of subjects without a systemic failure of sympathetic function the quantity and quality of sweat appears to depend also on other determinants, including but not limited to Na^+^ intake and/or balance, RAAS, age and physical activity. A relevant example comes from cystic fibrosis (CF), characterized by defective CFTR but also to secondary failure to activate ENaC and reabsorb Na^+^ [59]. Not only are CF homozygous individuals known to have low blood pressure [60] but also CF carriers were found to have lower systolic and diastolic pressures than matched control subjects, with a tendency for blood pressure to increase less with age [61]. Our HHS obese subjects could feature the opposite end of a spectrum.

This salt-retaining, water-losing phenotype of HHS subjects links to the last findings of our study: despite the lack of association with many clinical characteristics, the amount of TEWL measured at rest correlates with sweat volume and with surrogates of the skin microvascular status, i.e., circulating VEGF-c and Hb in the skin. In a recent study on psoriatic animals, prone to skin water loss from their disease plaques, TEWL was maintained similar to control animals in resting conditions by cutaneous vasoconstriction, paralleled by increased arterial blood pressure. However, external heating induced vasodilation and unveiled excess TEWL [13]. Other stressors, like physical activity, are likely to induce similar effects. In the multifactorial pathogenesis of obesity-associated hypertension [62], the skin could possibly contribute not only via disproportionate Na^+^ retention but also via excess water loss and the associated skin microvascular dysfunction, that we detected after cholinergic local stimulation and that other groups previously reported [63–67].

Our study has limitations, but also some strengths. First, its cross-sectional, non-interventional design limits our findings to associations and cannot directly inform on causality or long-term BP control in patients. In addition, antihypertensive therapy was not standardized, thus providing limited information on the effects of different drug classes and precluding the reliable assessment of renin and aldosterone in our patients. However, the study included a considerable number of participants, with a broad age range, who were fully representative of an unselected real-life cohort of hypertensive patients. Moreover, all measures of skin Na^+^ and water balance were investigated in relation to measures of tissue Na^+^ content, in addition to other circulating markers and clinical characteristics: we recognize that [Na^+^]_skin_ from a single punch biopsy is an imperfect surrogate for total body Na^+^, but the latter is impractical to measure and differences in skin Na^+^ were previously shown to parallel those in other organs [3], NTproBNP [3] and ^23^Na-MRI signal [45]. Although we lack a normotensive control group for quantitative comparison, this would not significantly add to the associations identified and their pathophysiological significance: our hypotheses-generating findings should inform interventional studies investigating the potential for therapeutic modulation of these local skin axes; these studies should aim for inclusion of ethnicities other than Caucasian, herein unrepresented. Additional limitations of our study are the regional and unphysiological, pilocarpine-mediated assessment of sweat parameters, or the resting-only measurement of TEWL: future studies in hypertensive patients should consider including physical activity as a physiological stressor. Nevertheless, our cholinergic stimulation of the skin already reveals important aspects of glandular and vascular function that can inform these next investigations.

## Conclusions

In conclusion, in this pragmatic study of a real-life cohort of hypertensive patients, we found that measures of Na^+^ and water skin handling are associated with clinically relevant characteristics, systemic Na^+^ status and blood pressure control. These findings suggest a potential role of the skin in body-fluid homeostasis. Importantly, these mechanisms may be amenable to therapeutic targeting, by approaches that include but are not limited to physical activity [68–72]. For their personalized implementation, the individual responses and the exact clinical/tissue predictors of response remain to be identified.

## Clinical perspectives

- Increasing evidence suggests excess skin Na^+^ accumulation in hypertension, but skin-specific mechanisms of local Na^+^ and water regulation in relation to body fluid and blood pressure homeostasis have largely been ignored.
- In a real-life cohort of hypertensive patients, we found that measures of Na^+^ and water skin handling are associated with relevant clinical characteristics, systemic Na^+^ status and blood pressure control.
- These findings suggest a possible role of the skin in body-fluid homeostasis in human health and disease, along with potential for therapeutic targeting of sweat rates/patterns and trans-epidermal water loss.

## Supplementary Material

Supplementary Figures S1-S2 and Tables S1-S2

## Data Availability

All source data supporting the findings of this study are available at: http://doi.org/10.25430/researchdata.cab.unipd.it.00000700.

## Abbreviations

BMI: body mass index
CCB: calcium channel blockers
DBP: diastolic blood pressure
HR: heart rate
HTN: hypertension
MRA: mineralocorticoid antagonists
RAAS: renin–angiotensin–aldosterone system
TWEL: trans-epidermal water loss
VEGF-c: vascular endothelial growth factor-c
