# Supplementary material for: Skin-specific mechanisms of body fluid regulation in hypertension
Source: Clin Sci (Lond). 2023 Feb 7;137(3):239–50. doi: 10.1042/CS20220609 (PMC10621731; doi:10.1042/CS20220609)
Supplement: Supplementary Figures S1-S2 and Tables S1-S2 [file CS-2022-0609_supp.pdf]

Skin-specific mechanisms of body fluid regulation in hypertension

SUPPLEMENTAL MATERIAL

Jun Yu Chen[1], Khai Syuen Chew[1], Sheon Mary[1], Philipp Boder[1], Domenico Bagordo[2], Gian Paolo Rossi[2], Rhian Margaret Touyz[3], Christian Delles[1], Giacomo Rossitto[1,2]

[1] School of Cardiovascular & Metabolic Health, University of Glasgow, UK

[2] Emergency Medicine and Hypertension, DIMED; Università degli Studi di Padova, Italy

[3] Department of Family Medicine, McGill University, Montreal, Canada.

Corresponding author:

Dr Giacomo Rossitto,

Emergency Medicine and Hypertension, DIMED, Università degli Studi di Padova,

Via Giustiniani 2, 35128 Padova, Italy

and

School of Cardiovascular & Metabolic Health, University of Glasgow

126 University Place, G12 8TA Glasgow, UK

e-mail: giacomo.rossitto@unipd.it

Suppl. Figure 1

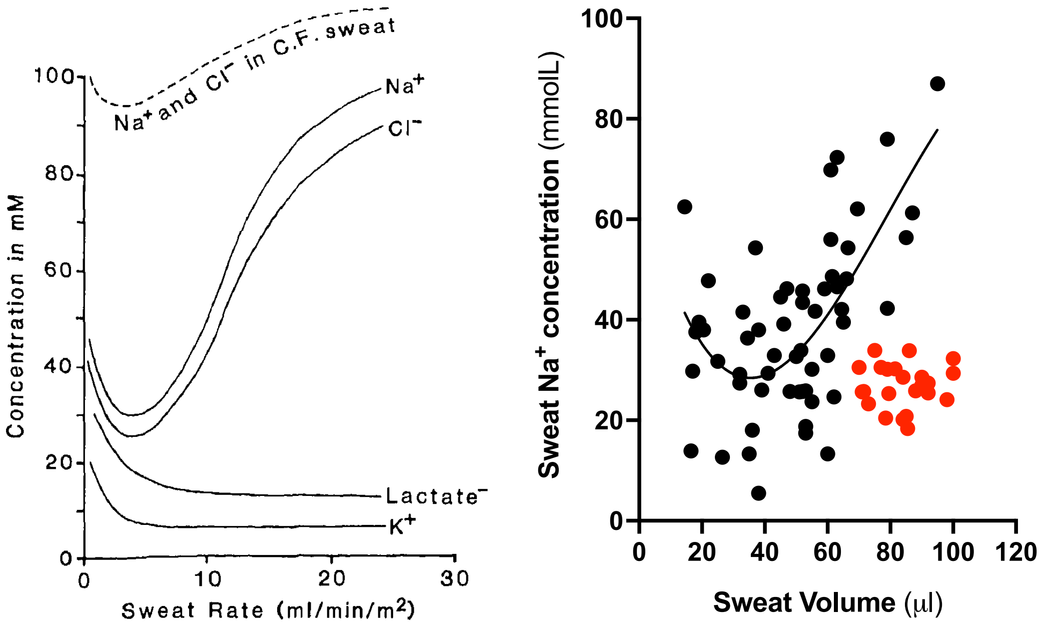

Relationship between sweat Na^{+} concentration and sweat volume in physiology (left; from *Sato K, J Am Academy Dermatology 1989, with permissions*) and in our hypertensive cohort (right). With the exception of a group showing high sweat volumes but low tonicity (red dots), a third order polynomial consistent with the physiological distribution also fit ($p<0.0005$) our study participants.

Suppl. Figure 2

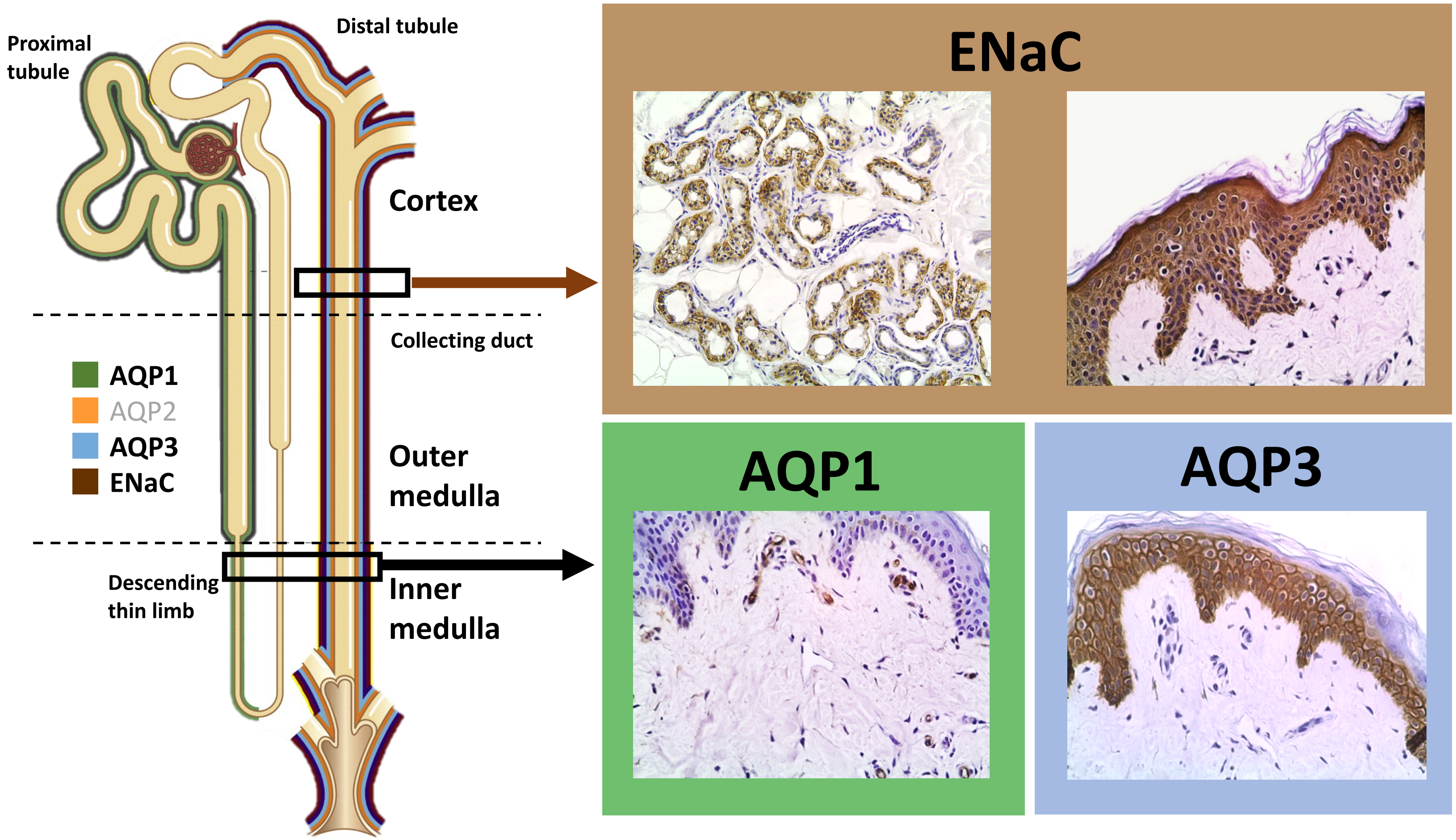

Representative immunohistochemical pictures from our lab from young healthy subjects, showing eccrine sweat gland and epidermal/microvascular staining for channels relevant to Na^+ and water handling, traditionally investigated in the kidney (left panel adapted from *Kortenoeven MLA and Fenton RA, Biochim Biophys Acta. 2014,* with permissions)

Suppl. Table 1. Correlations between sweat volume and composition with relevant covariates

Variables	Sweat Na^+ (mmol/l)		Sweat K^+ (mmol/l)		Sweat volume (µl)		TEWL	
Spearman	ρ	Sig	ρ	Sig	ρ	Sig	ρ	Sig
**Sex** (1M, 2F)	0.059	0.606	-0.016	0.888	-0.087	0.442	-0.163	0.132
**Age** (years)	0.341	**0.002**	-0.184	0.104	-0.167	0.139	-0.161	0.137
**BMI** (kg/m^2)	-0.086	0.456	-0.163	0.155	0.163	0.152	0.056	0.611
**Office SBP** (mmHg)	0.080	0.492	-0.167	0.146	-0.093	0.416	-0.156	0.154
**Office DBP** (mmHg)	-0.286	**0.012**	0.008	0.945	-0.028	0.809	-0.087	0.427
**Pulse pressure** (mmHg)	0.229	**0.045**	-0.254	**0.026**	-0.092	0.421	-0.153	0.163
**Na^+ intake** (questionnaire)	-0.033	0.776	-0.192	0.099	0.021	0.860	-0.132	0.284
**Epidermal Na^+** (mmol/l)	0.302	**0.019**	-0.055	0.677	-0.158	0.225	-0.132	0.284
**s-Na^+** (mmol/l)	0.091	0.442	-0.139	0.237	-0.095	0.420	-0.014	0.897
**s-K^+** (mmol/l)	-0.028	0.812	-0.186	0.110	0.117	0.312	0.035	0.758
**s-Urea** (mmol/l)	0.085	0.470	-0.049	0.680	-0.018	0.876	-0.091	0.414
**s-Creatinine** (umol/l)	-0.091	0.438	0.014	0.907	-0.020	0.865	-0.002	0.982
**NT-pro-BNP** (pg/ml)	0.087	0.461	-0.072	0.541	-0.085	0.467	0.060	0.589
**s-VEGFc** (pg/ml)	-0.076	0.557	-0.086	0.508	0.292	**0.020**	0.261	**0.029**

Sweat Na^+ = sweat sodium. Sweat K^+ = sweat potassium. TEWL = transepidermal water loss. BMI = Body Mass Index. SBP = systolic blood pressure. DBP = diastolic blood pressure. Pulse pressure = systolic blood pressure – diastolic blood pressure. Na^+ intake sodium intake score from food questionnaire. Sweat Na^+ = sweat sodium. Sweat K^+ = sweat potassium. Epidermal Na^+ = epidermal sodium. s- = serum. u-ACR = urinary albumin to creatinine ratio (random urine sample). NT-pro-BNP = N-terminal-pro B-type natriuretic peptide. s-VEGFc = serum vascular endothelial growth factor.

Suppl. Table 2. Characteristics of patients with controlled and uncontrolled blood pressure.

Variables	CONTROLLED (26.1%)	p	UNCONTROLLED (73.9%)
**Females**	16		26
**Age** (years)	49 ± 16	0.392	59 ± 15
**BMI** (kg/m^2)	29.8 (25.8-35.1)	0.580	29.7 (27.4-35.2)
**BMI class**			
**Normal weight**	3 (13%)		7 (10.8%)
**Overweight**	9 (39.1%)	0.951	27 (41.5%)
**Obese**	11 (47.8%)		31 (47.7%)
**Office SBP** (mmHg)	**128 ± 10**	**0.060**	**155 ± 18**
**Office DBP** (mmHg)	**82 ± 6**	**0.053**	**91 ± 12**
**Office HR** (beats per minute)	71 (65-87)	0.816	77 (65-87)
**Number of anti-HTN medications**	1 (0-2)	0.125	2 (1-2)
**0**	8 (34.8%)		14 (21.5%)
**1**	6 (26.1%)		16 (24.6%)
**2**	8 (34.8%)	0.276	19 (29.2%)
**3**	1 (4.3%)		11 (16.9%)
**≥ 4**	0 (0%)		5 (7.7%)
**ACEi/ARB**	15 (65.2%)	0.207	51 (78.5%)
**CCB**	10 (43.5%)	0.170	39 (60%)
**Diuretic**	8 (34.8%)	0.412	29 (44.6%)
**BB**	4 (17.4%)	0.397	17 (26.2%)
**MRA**	1 (4.3%)	0.169	10 (15.4%)
**AB**	2 (8.7%)	0.271	12 (18.5%)
**Obesity**	12 (52.2%)	0.908	33 (50.8%)
**Diabetes mellitus**	2 (9.1%)	0.562	9 (13.8%)
**Dyslipidaemia**	7 (31.8%)	**0.015**	39 (61.9%)
**eGFR<60** (mL/min/1.73 m2)	1 (4.3%)	0.217	9 (13.8%)
**Na$^+$ intake** (g/d; questionnaire)	2.62 (1.99-3.28)	0.588	2.87 (2.08-3.73)
**s-Na$^+$** (mmol/l)	140 (139-141)	0.469	140 (139-142)
**s-Urea** (mmol/l)	5.0 (4.0-5.9)	0.297	5.2 (4.6-6.7)
**s-Creatinine** (umol/l)	69 (60-76)	0.098	76 (66-87)
**NT-pro-BNP** (pg/ml)	52.3 (29.7-118.7)	0.071	80.2 (44.7-224.7)
**s-VEGFc** (ng/ml)	14.6 ± 2.5	**0.019**	12.7 ± 3.4

Qualitative data presented as n (%). Quantitative data presented as mean ± SD or median (interquartile range), as appropriate. BMI = Body Mass Index. BMI class: 1 = underweight (<18.5), normal weight (18.5 - 24.9), 2 = overweight (25.0–29.9), 3 = obesity (>= 30). SBP = systolic blood pressure. DBP = diastolic blood pressure. HR = heart rate. HTN = hypertension. Uncontrolled HTN = SBP ≥ 140 and/or DBP ≥ 90 mmHg. ACEi/ARB = ACE inhibitors or Angiotensin receptor blockers. CCB = calcium channel blockers. BB = beta blockers. MRA = mineralocorticoid antagonists. AB = alpha blockers.
